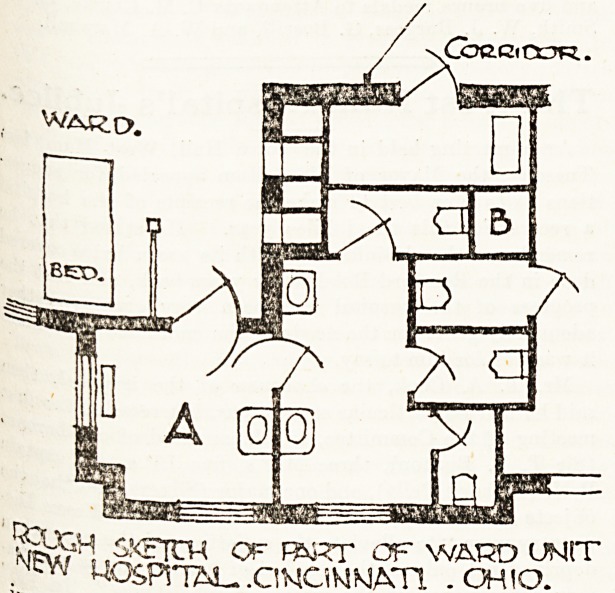# The Disconnection of Sanitary Towers

**Published:** 1912-05-25

**Authors:** 


### HOSPITAL ARCHITECTURE AND CONSTRUCTION.

[Communications on this subject should be marked "Architecture" in the left-hand top corner of the envelope.]

### The Disconnection of Sanitary Towers.

Modern hospital construction requires that the sanitary annexe shall be efficiently cut off from the ward unit by a ventilated area. The same principle of design may be found in the building laws of most authorities, which require that no water-closet shall be entered direct from a living room. The most efficient method of providing this "cut-off" in the construction of a hospital is to provide a bridge or gangway of sufficient width, probably 6 ft., and of as low a height as is compatible with efficiency, say 7 ft. 6 in. in the clear. This should be constructed of glazed brick walls with continuous hardwood windows in the upper part and with steel and concrete floor and roof, the former finished in tiles or mosaic with a cavetto skirting. The position of the sanitary tower in relation to the aspect of the ward will determine the length of the bridge, for it is most

ROUGH SKETCH OF PART OF WARD UNIT NEW HOSPITAL. CINCINNATI. OHIO.

imperative that shadows projected on to the ward windows be at all times avoided. In a case where the axial line of the wards is due north and south and the annexes are placed to the east and west, the length of this bridge might be about 6 ft., which, with double doors 5 ft. wide at each end, would give ample passing room. If the height of each superimposed ward is, say, 12 ft. 6 in., there would be an air-space between the roof of one gangway and the floor of the next above of, say, 5 ft. 6 in., which would ensure a continuous fresh-air current between one and the other, and further serves the purpose of ensuring a continual movement of the air about the ward walls, as if this flow through did not exist there might be a tendency to stagnation of the atmosphere against the bridge ways. Some architects favour the practice of placing a wheel bath in the disconnecting corridor, and others favour this situation for the erection of sanitary cupboards, which, even though well ventilated, are better omitted. The only item which should find a place in the bridge is a radiator to avoid chilling to patients passing from the tempered air of the ward.

When the position of the sanitary tower is such in its relation to the ward that a staircase for escape in cases of panic can be placed leading from the bridge or gangway to the ground level without obstructing the ward windows, it is an ideal position. For there is only one greater factor, and that is that there should be sufficient room to get a staircase in short flights with an easy gradient and with wide landings, to facilitate the easy carrying of patients in beds or on stretchers. Of the type of window most suitable for the bridge to ensure just a sufficient amount of fresh air, probably the "Austral," described in our issue of April 13, is best, and should be of a double-sash type.

The plans of the winning design in the Bradford Royal Infirmary competition show the sanitary towers in the form of an octagon, as if the architect wished to avoid cutting off the light and air more than is absolutely essential. Indeed American architects favour a style of planning in which the sanitary tower, as we understand the term in this country, is not disconnected, but forms an integral part of the pavilion; the objection of shadows cast on the ward wall, as before stated, is therefore at once eliminated. The accompanying rough sketch of the New General Hospital, Cincinnati, Ohio, for which Messrs. Hannafords were architects, illustrates the point. It will be seen that a double system of disconnection here obtains. First, the sanitary offices are isolated by the chamber A on plan, which is 6 ft. 6 in. square, and contains only a radiator and a washhand basin for the use of the medical officer, and secondly, the vitiated air from the waterclosets is drawn into an extraction shaft carried up to the top of the roof, and rendered efficient by a powerful fan extract. The sanitary block is some 8 ft. wide and about 13 ft. 6 in. long, and contains four pedestals, one being for staff use and approached separately; it also contains two washbasins. The pedestal seats are weighted, so that normally they stand vertical. No urinals are provided, it being found in America, as in this country, that a pedestal such as described combines the same utility with less offence.

The point B on the plan is the vent shaft, to which a parallel may be found in a leading London hotel,* where the sanitary blocks are all ventilated into such chambers or shafts which, while serving their purpose, give also accommodation for the numerous pipes and conduits inseparable from such an establishment. The great advantage of this system of planning is that it precludes the necessity of providing a separate window to each water-closet, and that is an important consideration with the designer in the planning of the sanitary arrangements for the ward unit of the general hospital.

* The Strand Palace Hotel; Mr. W. J. Ancell, Architect.

## Figures and Tables

**Figure f1:**